# Supplementary material for: Mitotic DNA synthesis in response to replication stress requires the sequential action of DNA polymerases zeta and delta in human cells
Source: Nat Commun. 2023 Feb 9;14:706. doi: 10.1038/s41467-023-35992-5 (PMC9911744; doi:10.1038/s41467-023-35992-5)
Supplement: Supplementary file 1 — Supplementary information [file 41467_2023_35992_MOESM1_ESM.pdf]

Supplementary Information

Mitotic DNA synthesis in response to replication stress requires the sequential action of DNA polymerases zeta and delta in human cells

Wei Wu, Szymon A. Barwacz, Rahul Bhowmick, Katrine Lundgaard, Marisa M. Gonçalves Dinis, Malgorzata Clausen, Masato T. Kanemaki and Ying Liu

Supplementary figures and legends 1 to 12

Supplementary Table 1 (Reagents table)

FACS gating strategy

Supplementary Fig.1

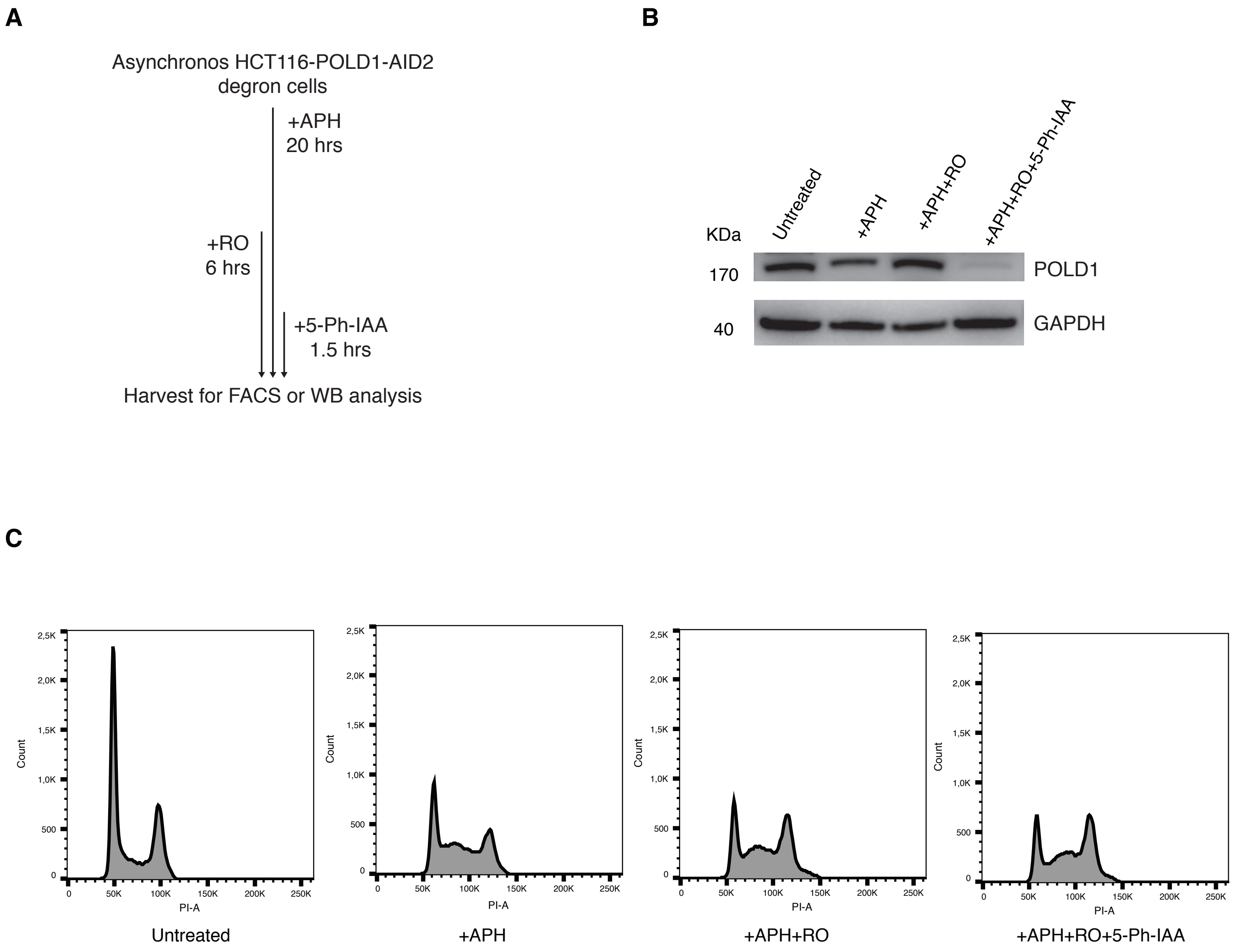

Supplementary Fig. 1: HCT116-POLD1-AID2 cells were accumulated at G2 phase following RO3306 treatment.

(**A**) Experimental workflow for cell synchronization and flow cytometry (FACS) or Western blot (WB) of analysis of HCT116-POLD1-AID2 cells following treatment with 5-Ph-IAA and RO3306 (RO). (**B**) Western blot analysis of POLD1 expression at the end of the treatment. GAPDH was used as a loading control. (**C**) Representative histogram of propidium iodide (PI) fluorescence of HCT116-POLD1-AID2 cells harvested at end of the treatment. Cells were fixed with 70% ethanol, stained with propidium iodide, and analyzed by flow cytometry. Untreated cells were used as a control. X-axis, total PI area; Y-axis, cell count.

Supplementary Fig. 2

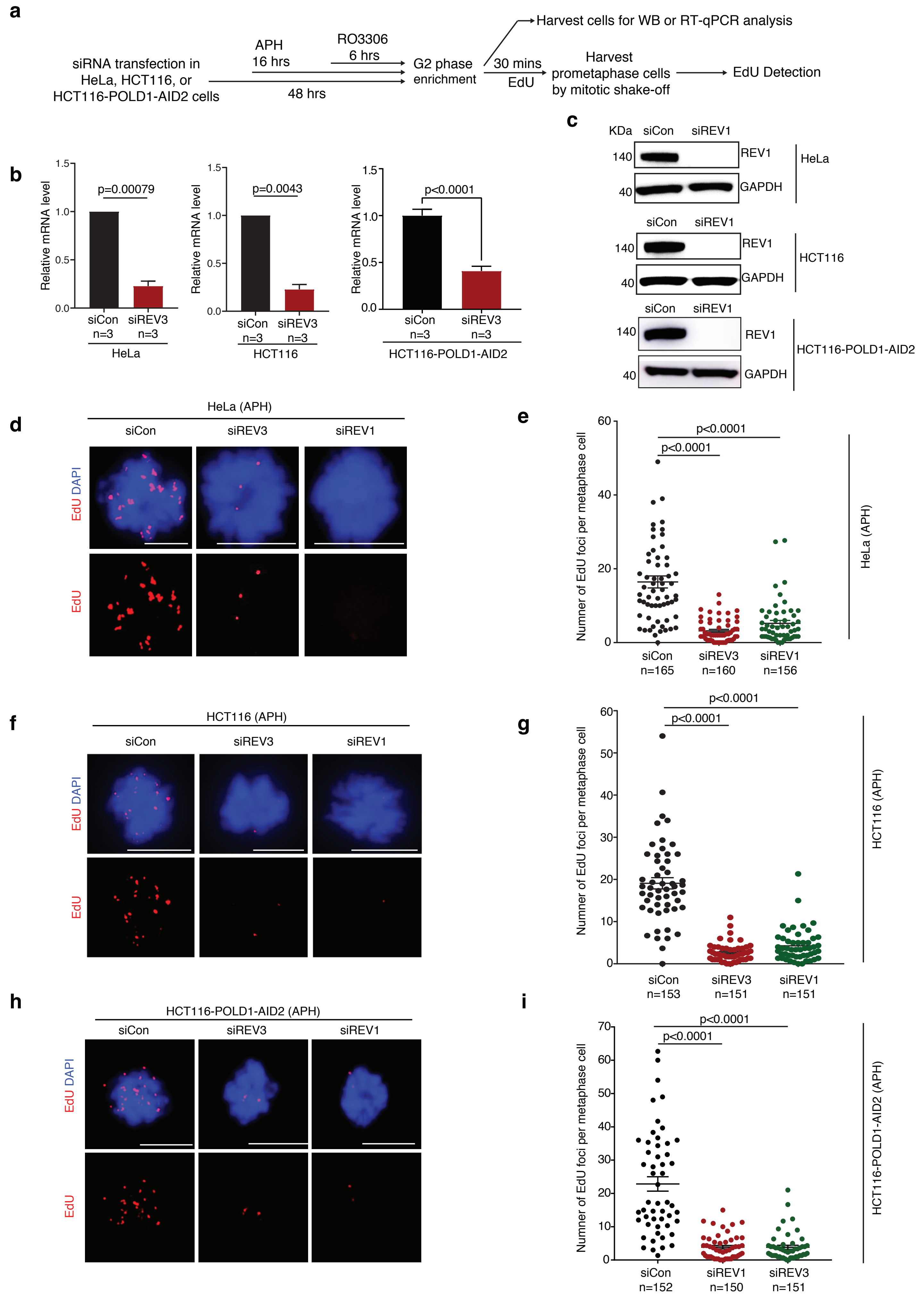

Supplementary Fig. 2: REV3 and REV1 are essential for MiDAS in HeLa and HCT116 cells.

a Experimental workflow of RT-qPCR and Western blotting analysis with HeLa, HCT116 or HCT116-POLD1-AID2 cells following REV3 or REV1 depletion and APH treatment (0.4 μM). **b** Quantification of REV3 mRNA by RT-qPCR after transfecting the cells with control siRNA or REV3 siRNA. The RT-qPCR value was normalized against a region of the GAPDH gene for each sample. Data of each bar are means of independent experiments. Error bars represent SEM. P values were calculated using a two-tailed Student's t-test (n=3 biological replicates). **c** Western blot analysis of REV1 after transfecting cells with control siRNA or REV1 siRNA. GAPDH was used as a loading control. Representative images (**d**) and quantification (**e**) of MiDAS foci (labeled with EdU; red) in prometaphase HeLa cells treated as shown in panel a. Representative images (**f**) and quantification (**g**) of MiDAS foci (labeled with EdU; red) in prometaphase HCT116 cells treated as shown in panel a. Representative images (**h**) and quantification (**i**) of MiDAS foci (labeled with EdU; red) in prometaphase HCT116-POLD2-AID2 cells treated as shown in panel a. DNA was stained with DAPI (blue). Each data point in charts of e, g, i is means of three independent experiments and plotted with Prism (n= number of cells analyzed in each condition in three experiments). Error bars represent SEM. P values were calculated using a two-tailed non-parametric Mann–Whitney test. Scale bars, 10 μm. Hr, hour; min, minute.

Supplementary Fig. 3

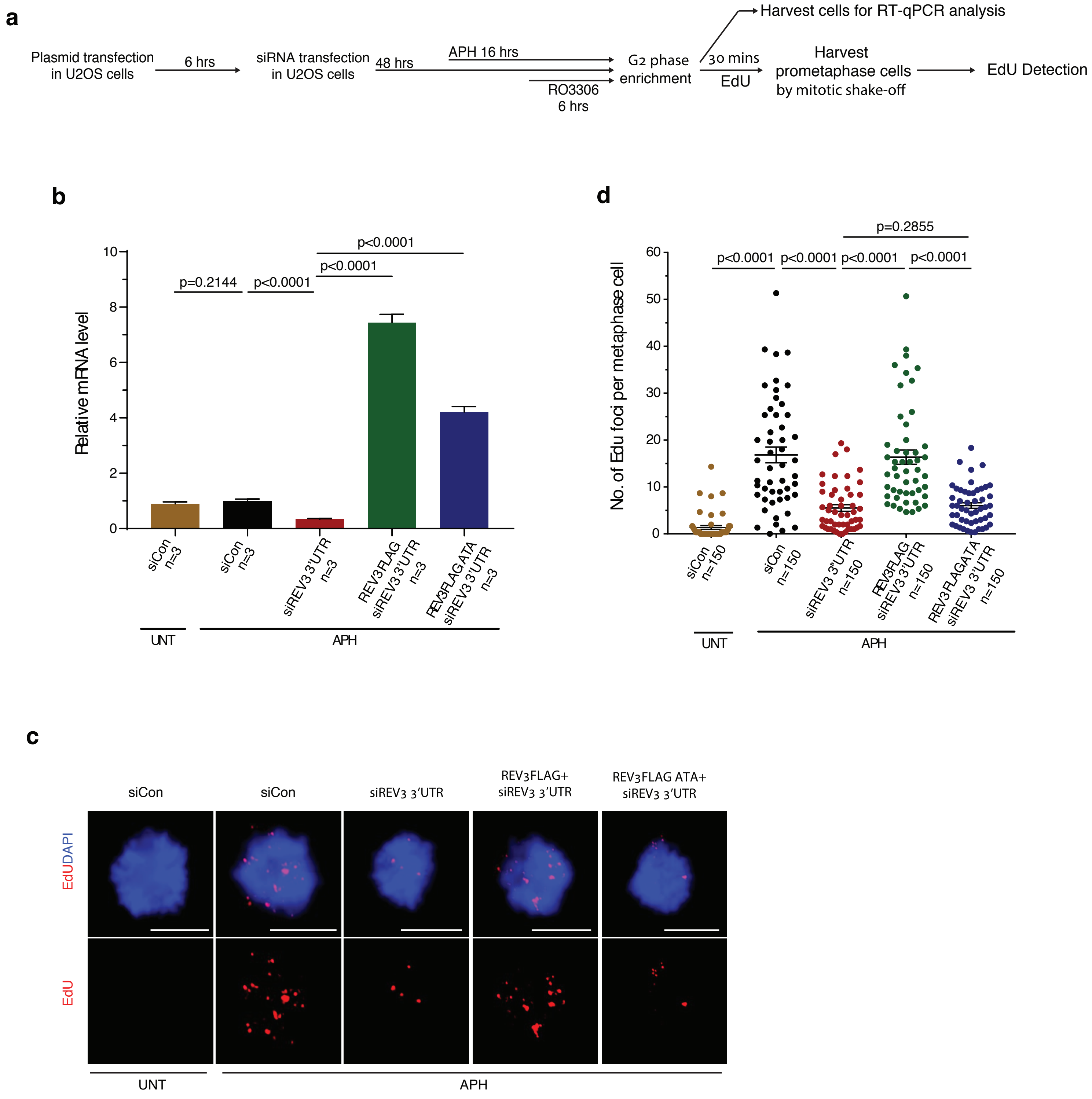

Supplementary Fig. 3: The catalytic domain of REV3 plays a role in MiDAS.

a Experimental workflow for MiDAS analysis in U2OS cell transfected with a REV3-FLAG or a REV3-FLAGATA mutant plasmid, and then treated with control or an siRNA targeting the 3' UTR of REV3 and APH (0.4 μM). Following 6 hours RO3306 treatment, cells were rinsed three times with pre-warmed, drug-free culture medium within 5 minutes, and then incubated with fresh media with EdU for 30 minutes before MiDAS analysis. **b** Quantification of REV3 mRNA using RT-qPCR. The RT-qPCR value was normalized against a region of the GAPDH gene for each sample. Data of each bar are means of three independent experiments. Error bars represent SEM. P values were calculated using a two-tailed Student's t-test (n=3 biological replicates). Representative images (**c**) and quantification (**d**) of MiDAS (labelled with EdU; red). Each data point in chart d is mean of three independent experiments and plotted with Prism (n= number of cells analyzed in each condition in three experiments). Error bars represent SEM. P values were calculated using a two-tailed non-parametric Mann–Whitney test. Scale bars, 10 μm. Hr, hour; min, minute.

Supplementary Fig. 4

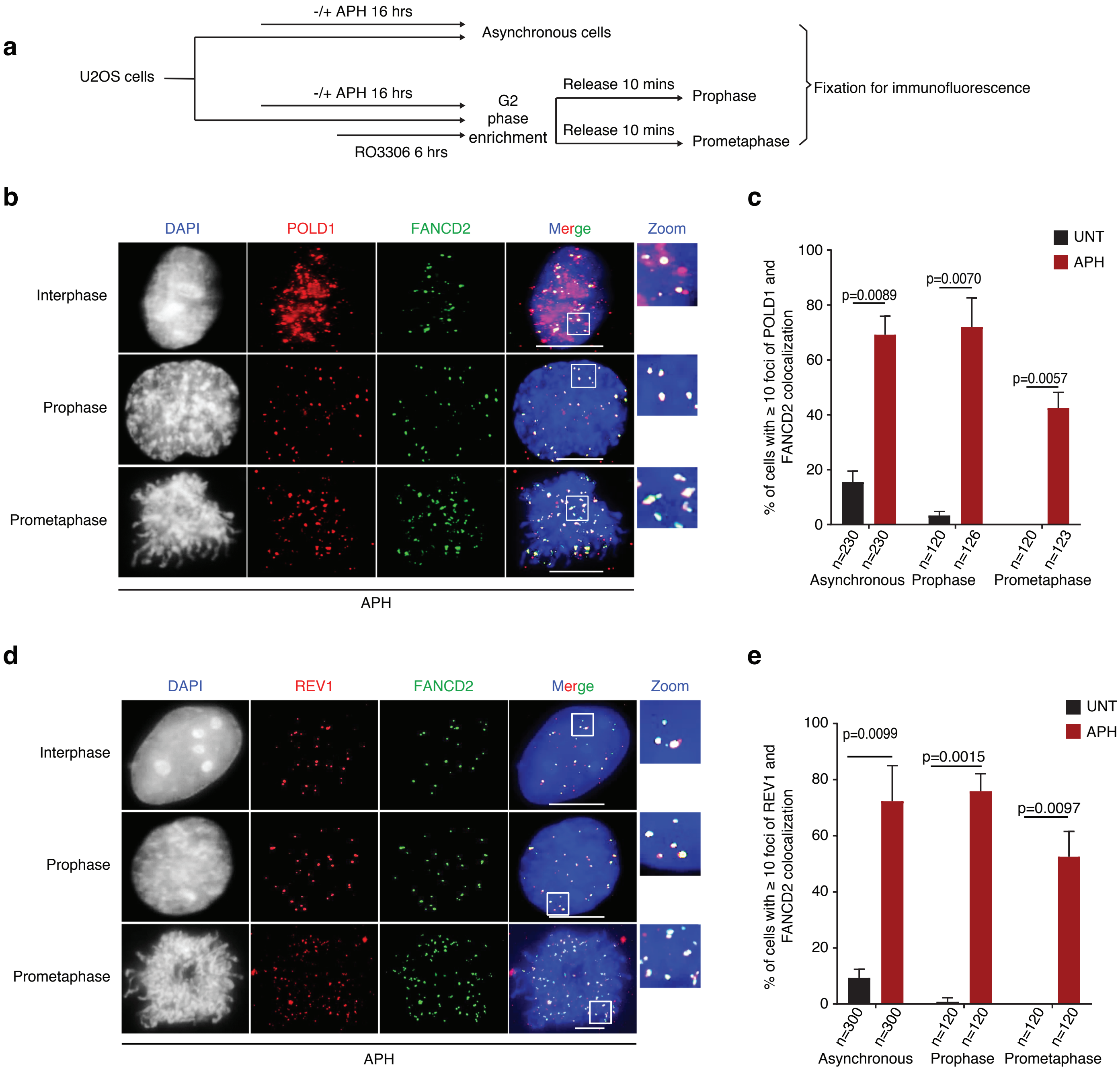

Supplementary Fig. 4: POLD1 co-localizes with REV1 and FANCD2 in mitosis when cells are challenged with RS.

a Experimental workflow for U2OS cell synchronization at prophase or prometaphase under untreated or APH treatment (0.4 μM) conditions. **b** Representative immunofluorescence images of interphase, prophase and prometaphase cells treated with APH (0.4 μM) for 16 hours and stained with POLD1 antibody (red) and FANCD2 antibody (green). DNA was stained with DAPI (blue). A zoomed image from the cell indicated by a white box is shown on the right. **c** Quantification of the co-localization of POLD1 foci with FANCD2 foci in cells treated as shown in panel a. **d** Representative immunofluorescence images of interphase, prophase and prometaphase cells stained with REV1 antibody (red) and FANCD2 antibody (green). DNA was stained with DAPI (blue). A zoomed image from the cell indicated by a white box is shown on the right. **e** Quantification of the co-localization of REV1 foci with FANCD2 foci in cells treated as shown in panel a. Data in charts c and e are means of three independent experiments and plotted with Prism (n= number of cells analyzed in each condition in three experiments). Error bars represent SEM. P values were calculated using a two-tailed Student's t-test. Scale bars, 10 μm. Hr, hour; min, minute.

Supplementary Fig. 5

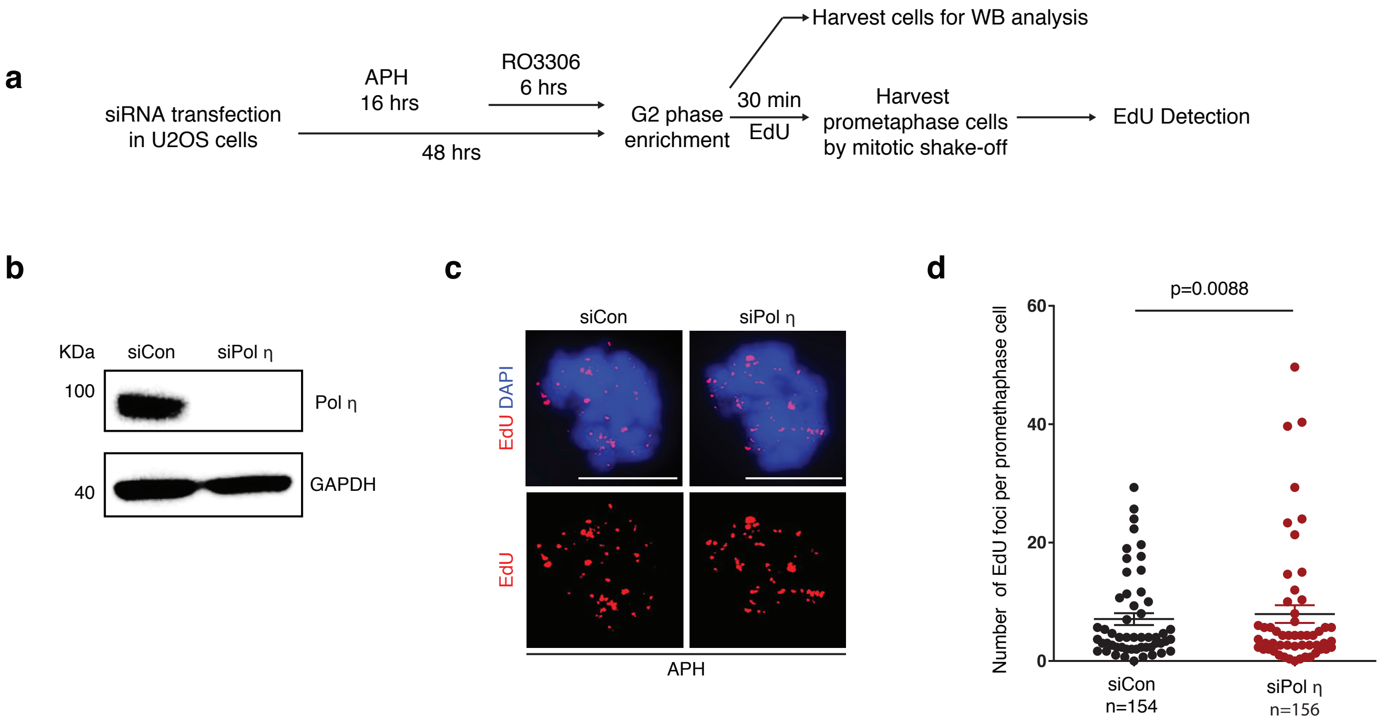

Supplementary Fig. 5: Pol η is not required for MiDAS.

a Experimental workflow for analysis of MiDAS in prometaphase U2OS cells following Pol η depletion and APH treatment (0.4 μM). Following 6 hours RO3306 treatment, cells were rinsed three times with pre-warmed, drug-free culture medium within 5 minutes, and then incubated with fresh media with EdU for 30 minutes before MiDAS analysis. **b** Western blot (WB) analysis of Pol η after transfecting cells with control or Pol η siRNAs. GAPDH was a loading control. Representative images (**c**) and quantification (**d**) of MiDAS foci (labeled with EdU; red) in prometaphase cells treated as shown in panel a. DNA was stained with DAPI (blue). Each data point in chart d is mean of three independent experiments and plotted with Prism (n= number of cells analyzed in each condition in three experiments). Error bars represent SEM. P values were calculated using a two-tailed non-parametric Mann–Whitney test. Scale bars, 10 μm. Hr, hour; min, minute.

Supplementary Fig. 6

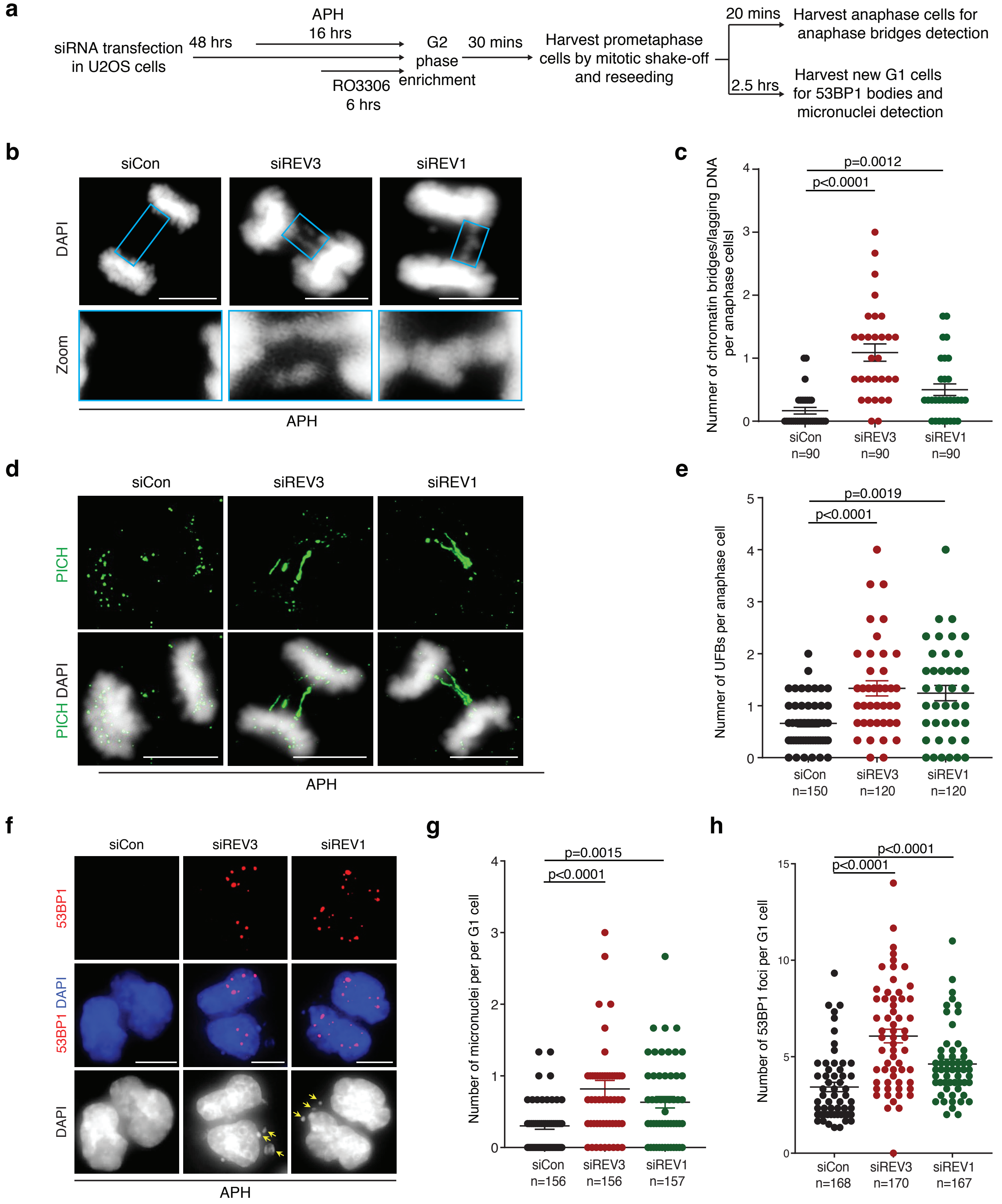

Supplementary Fig. 6: REV3 and REV1 are essential for genome stability.

a Experimental workflow for analyzing U2OS cells in metaphase, anaphase or in the next G1 phase following REV1 or REV3 depletion and APH treatment (0.4 μM). Following 6 hours RO3306 treatment, cells were rinsed three times with pre-warmed, drug-free culture medium within 5 minutes, and then incubated with fresh media for the follow-up steps. Representative images (**b**) and quantification (**c**) of DAPI-positive bridges or lagging chromatin in U2OS cells during anaphase following the treatment indicated in panel a. DNA was stained with DAPI. Areas marked by blue boxes are zoomed in the lower panel of b. Representative immunofluorescence images (**d**) and quantification (**e**) of UFBs (marked by PICH; green) in U2OS cells during anaphase following the treatment indicated in panel a. DNA was stained with DAPI. Representative immunofluorescence images (**f**) and quantification of micronuclei (yellow arrows) (**g**) or 53BP1 bodies (red) (**h**) in newly born U2OS G1 cells following the treatment shown in panel a. DNA was stained with DAPI. Each data point in charts c, e, g, and h is means of three independent experiments and plotted with Prism (n= number of cells analyzed in each condition in three experiments). Error bars represent SEM. P values were calculated using a two-tailed non-parametric Mann–Whitney test. Scale bars, 10 μm. Hr, hour; min, minute.

Supplementary Fig. 7

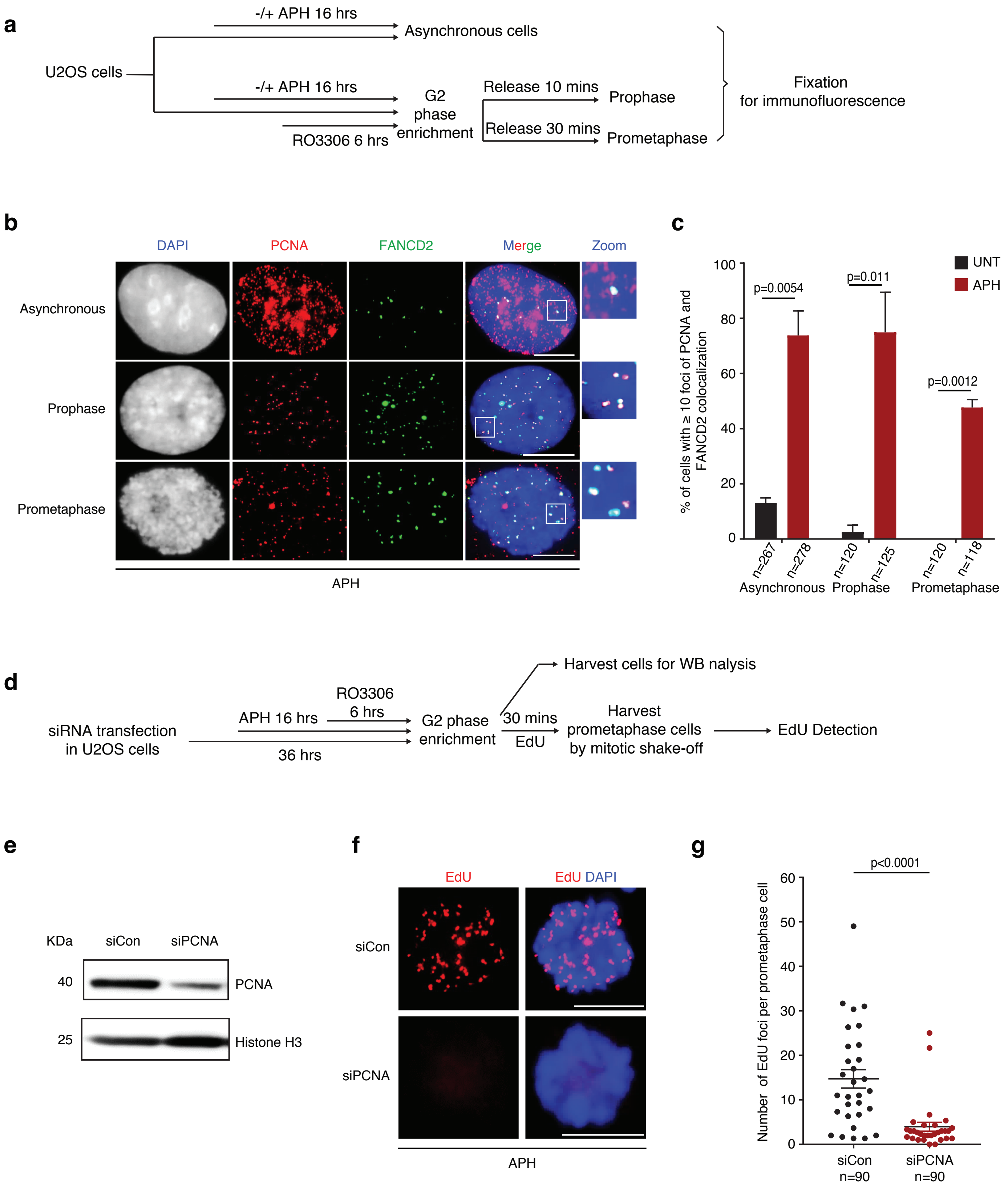

Supplementary Fig. 7: PCNA is required for MiDAS.

a Experimental workflow for immunofluorescence analysis (IF) of prophase or prometaphase cells following APH treatment (0.4 μM) treatment. Following 6 hours RO3306 treatment, cells were rinsed three times with pre-warmed, drug-free culture medium within 5 minutes, and then incubated with fresh media for the follow-up steps. **b** Representative immunofluorescence images of interphase, prophase and prometaphase cells treated with APH (0.4 μM) for 16 hours and stained with a PCNA antibody (red) or a FANCD2 antibody (green). DNA was stained with DAPI (blue). A zoomed image from the cell indicated by a white box is shown on the right. **c** Quantification of the co-localization of PCNA foci with FANCD2 foci in cells treated as shown in panel a. Data in chart are means of independent experiments (n= 3 biological replicates). Error bars represent SEM. P values were calculated using a two-tailed Student's t-test. **d** Experimental workflow for analysis of MiDAS in prometaphase cells following PCNA depletion and APH treatment (0.4 μM). Following 6 hours RO3306 treatment, cells were rinsed three times with pre-warmed, drug-free culture medium within 5 minutes, and then incubated with fresh media with EdU for 30 minutes before MiDAS analysis. **e** Western blot analysis of PCNA after transfecting cells with control PCNA siRNAs. Histone H3 was used as a loading control. Representative images (**f**) and quantification (**g**) of MiDAS foci (labeled with EdU; red) in prometaphase cells treated as shown in panel d. DNA was stained with DAPI (blue). Each data point in chart g is means of three independent experiments and plotted with Prism (n= number of cells analyzed in each condition in three experiments). Error bars represent SEM. P values were calculated using a two-tailed non-parametric Mann–Whitney test. Scale bars, 10 μm. Hr, hour; min, minute.

Supplementary Fig. 8

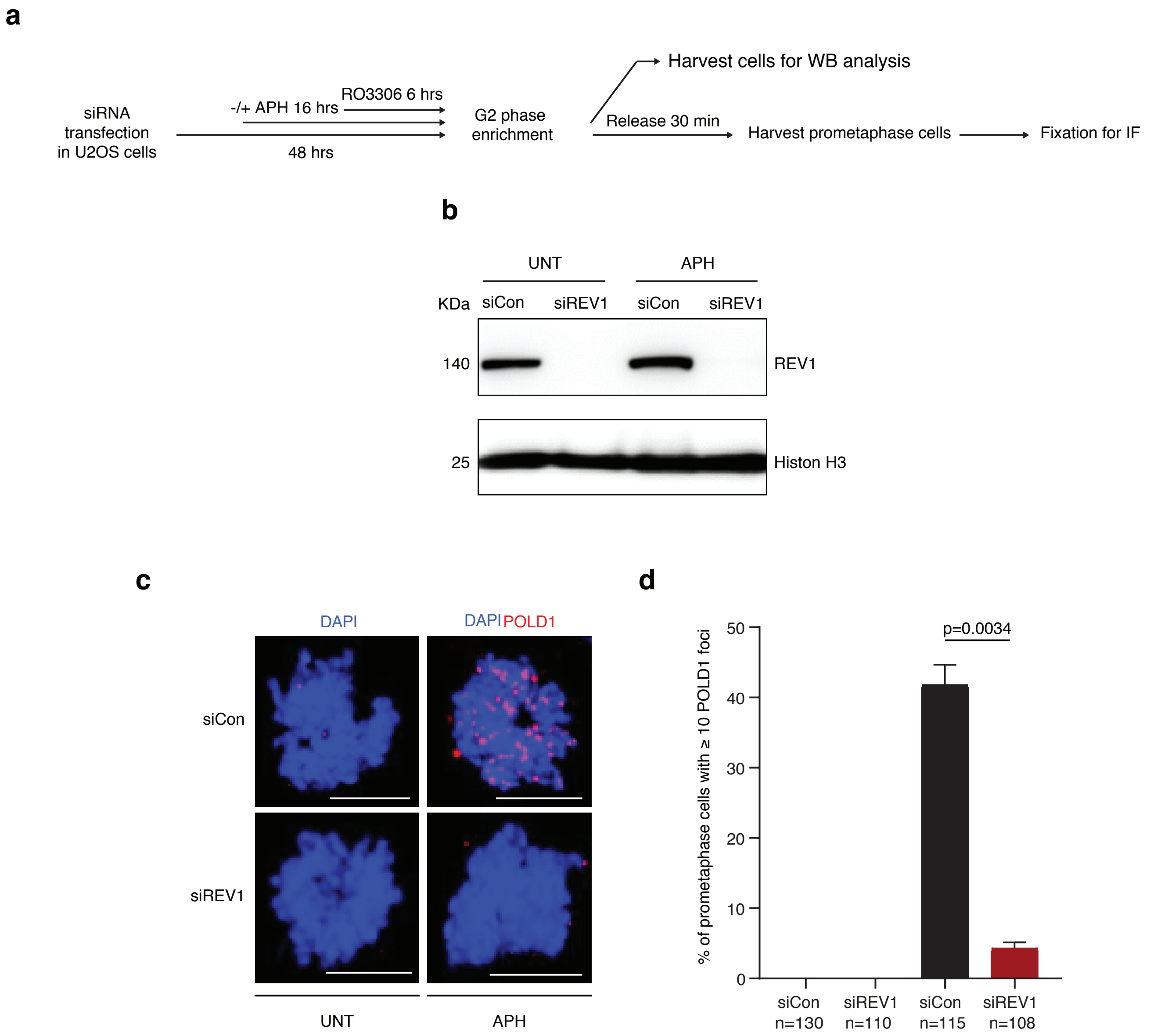

Supplementary Fig. 8. POLD1 recruitment in mitosis depends on REV1 when cells are challenged with RS.

a Experimental workflow for U2OS cells treated with siRNAs and then APH treatment (0.4 μM) for immunofluorescence (IF) analysis. Following 6 hours RO3306 treatment, cells were rinsed three times with pre-warmed, drug-free culture medium within 5 minutes, and then incubated with fresh media for 30 minutes followed by fixation and IF analysis. **b** Western blot (WB) analysis of REV1. Histone H3 was used as loading control. Representative IF images (**c**) and quantification (**d**) of POLD1 foci (red) in untreated or APH-treated prometaphase U2OS cells. DNA was stained with DAPI (blue). Data in chart d are means of three independent experiments and plotted with Prism (n= number of cells analyzed in each condition in three experiments). Error bars represent SEM. P values were calculated using a two-tailed Student's t-test. Scale bars, 10 μm. Hr, hour; min, minute.

Supplementary Fig. 9

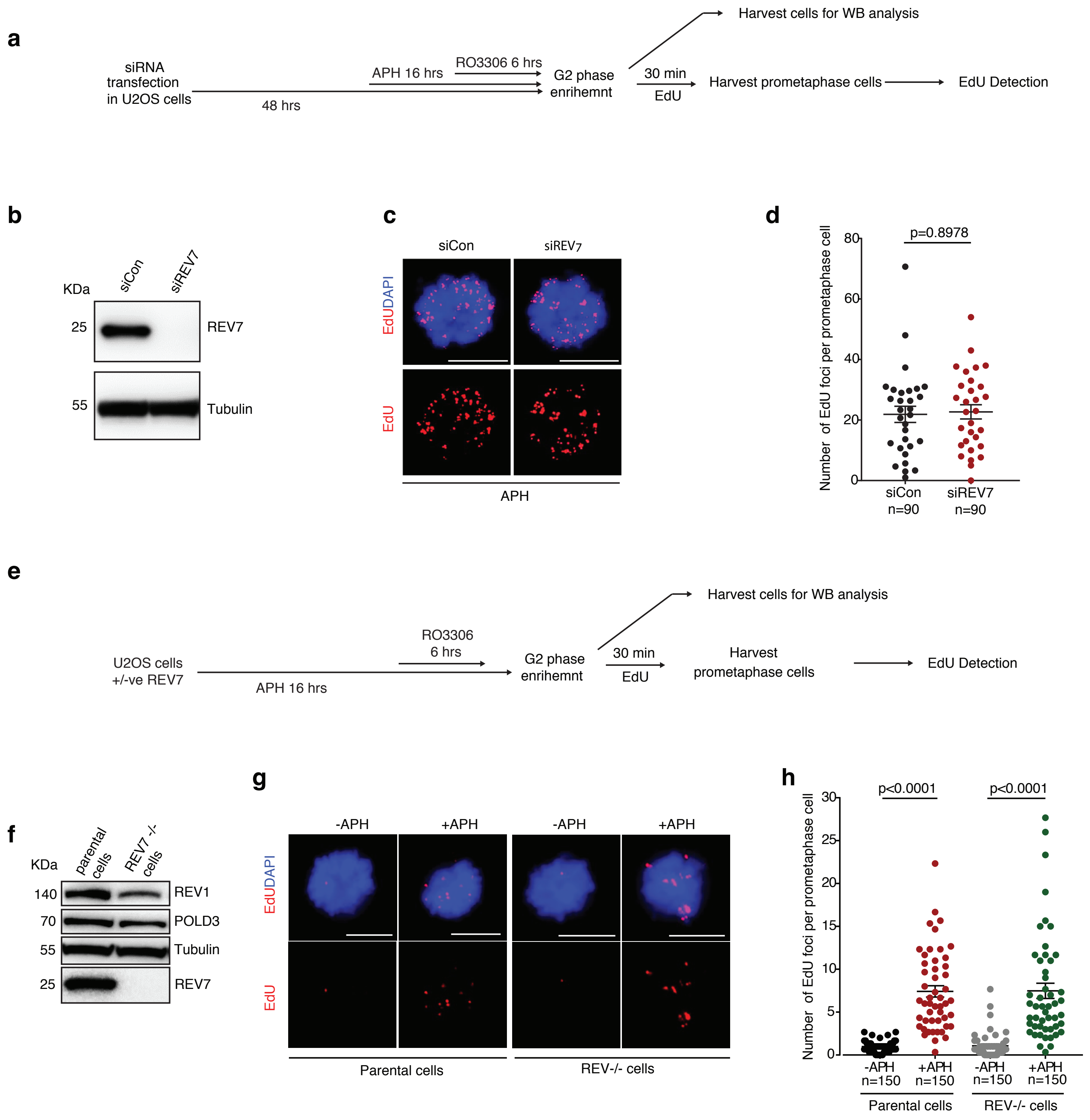

Supplementary Fig. 9: Loss of REV7 does not affect MiDAS.

a Experimental workflow for MiDAS analysis of U2OS cells following REV7 siRNA treatment and APH treatment (0.4 μM). Following 6 hours RO3306 treatment, cells were rinsed three times with pre-warmed, drug-free culture medium within 5 minutes, and then incubated with fresh media for 30 minutes followed by fixation and IF analysis. **b** Western blot analysis of REV7. β-tubulin was used as a loading control. Representative immunofluorescence images (**c**) and quantification (**d**) of MiDAS foci (labeled with EdU; red) in prometaphase cells. **e** Experimental workflow for MiDAS analysis in parental and $REV7^{-/-}$ U2OS cells following APH treatment (0.4 μM). Following 6 hours RO3306 treatment, cells were rinsed three times with pre-warmed, drug-free culture medium within 5 minutes, and then incubated with fresh media for 30 minutes followed by fixation and IF analysis. **f** Western blot analysis of REV7. β-tubulin was used as a loading control. The expression of REV1 and POLD3 were also examined in this assay. Representative immunofluorescence images (**g**) and quantification (**h**) of MiDAS foci (labeled with EdU; red) in prometaphase cells. DNA was stained with DAPI (blue). Each data point in charts d and h is means of three independent experiments and plotted with Prism (n= number of cells analyzed in each condition in three experiments). Error bars represent SEM. P values were calculated using a two-tailed non-parametric Mann–Whitney test. Scale bars, 10 μm. Hr, hour; min, minute.

Supplementary Fig. 10

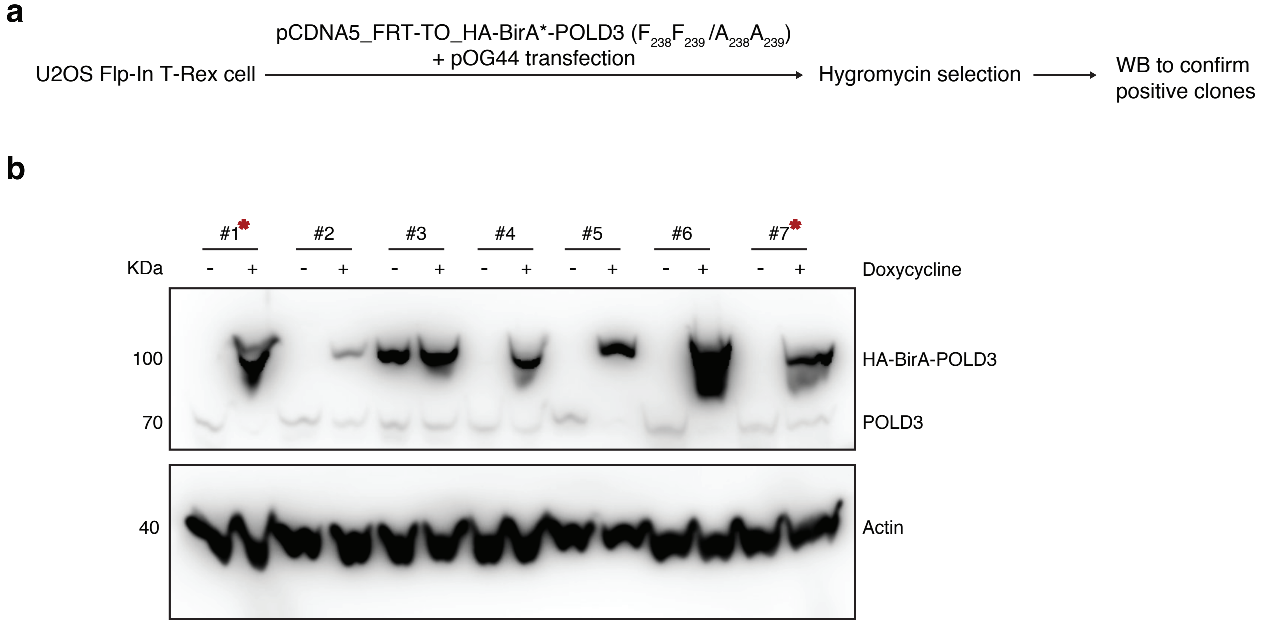

Supplementary Fig. 10: Establishment of U2OS cell clones with doxycycline inducible expression of wild type or mutant BirA-POLD3 cell lines.

a Workflow for the establishment of doxycycline inducible WT or mutant BirA-POLD3 ($F_{238}F_{239}A_{238}A_{239}$) (FF/AA) clones from U2OS Flp-In T-Rex parental cells. **b** Western blot analysis of BirA-POLD3 expression upon doxycycline induction in cell clones 1-7. Actin was used as a loading control. Red stars indicate the clones analyzed in the follow-up assays.

Supplementary Fig. 11

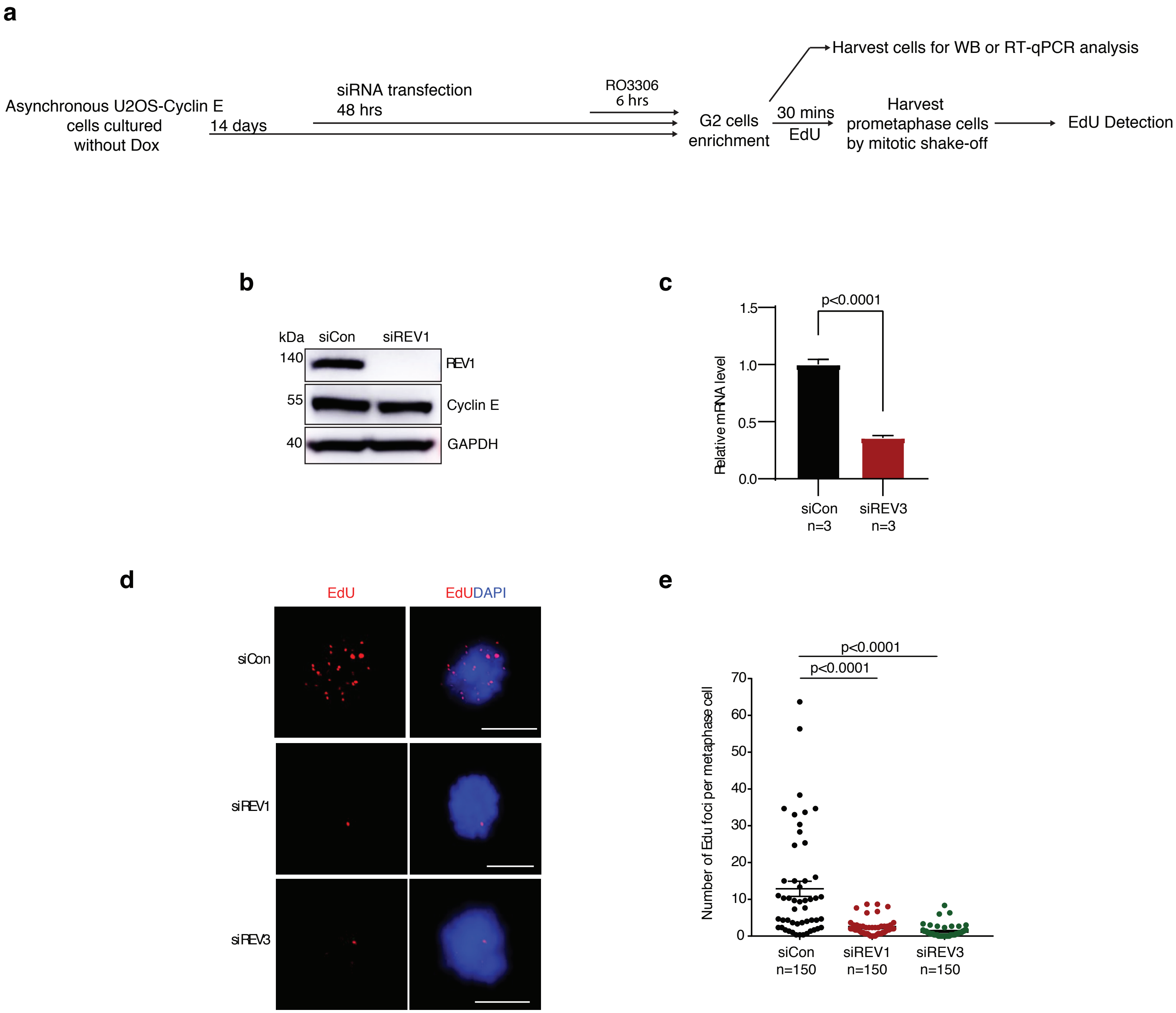

Supplementary Fig. 11: MiDAS in cells with overexpression of Cyclin E depends on REV1 or REV3.

a Experimental workflow for overexpression of Cyclin E, enrichment of cells at G2/M boundary and harvesting in prometaphase for MiDAS analysis. The expression of Cyclin E was activated by culturing cells without doxycycline for 14 days. Following 6 hours RO3306 treatment, cells were rinsed three times with pre-warmed, drug-free culture medium within 5 minutes, and then incubated with fresh media with EdU before MiDAS analysis. **b** Western blot analysis of REV1 and Cyclin E. GAPDH was used as a loading control. **c** Quantification of REV3 mRNA using RT-qPCRs. The RT-qPCR values were normalized against a region of the GAPDH gene for each sample. Data of each bar are means of independent experiments. Error bars represent SEM. P values were calculated using a two-tailed Student's t-test (n=3 biological replicates). Representative images (**d**) and quantification (**e**) of MiDAS foci (labeled with EdU; red) in prometaphase cells treated as shown in panel a. DNA was stained with DAPI (blue). Each data point in chart e is means of three independent experiments and plotted with Prism (n= number of cells analyzed in each condition in three experiments). Error bars represent SEM. P values were calculated using a two-tailed non-parametric Mann–Whitney test. Scale bars, 10 µm. Hr, hour; min, minute.

Supplementary Fig. 12

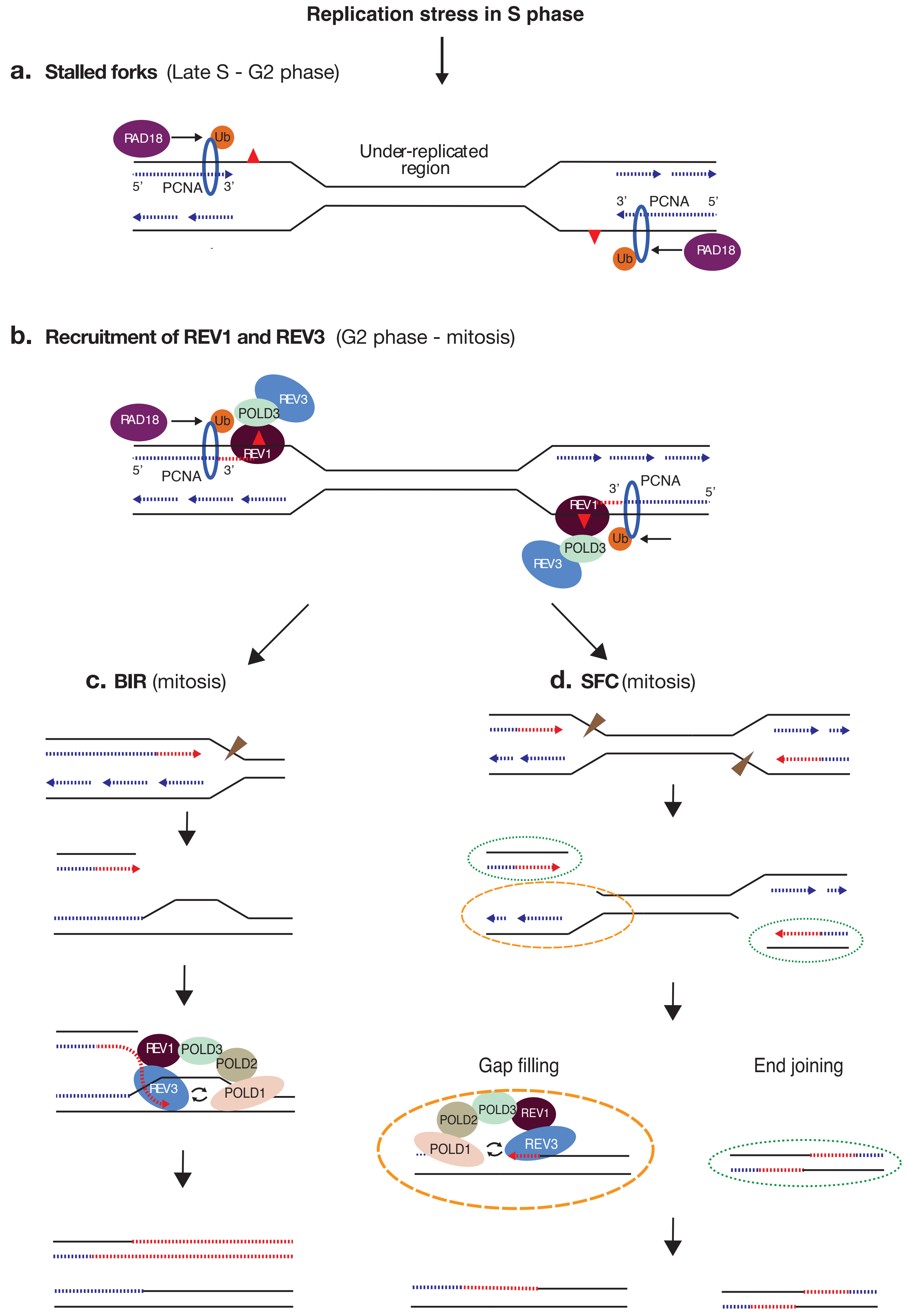

Supplementary Fig. 12: Proposed models for how TLS polymerases promote MiDAS.

a Following exposure to RS in S-phase, replication forks can become arrested or disrupted. This would activate a DNA damage response, leading to the monoubiquitylation of PCNA by RAD18. Black solid lines represent the parental DNA strands, while the nascent leading and lagging strands are shown by blue dotted lines. **b** Monoubiquitylated PCNA can recruit REV1, which might then act as a deoxycytidyl transferase and/or a scaffold protein for recruitment of other repair factors. Recruitment of REV3 and POLD3 by REV1 would permit the extension of the nascent DNA (red dotted lines). If replication of the locus were still incomplete when the cell entered mitosis, at least two strategies might be employed to rapidly process the stalled replication forks and conduct 'unscheduled' DNA synthesis (MiDAS) to finish locus duplication. **c** In the 'break-induced replication' model (BIR), the leading strand template would be cut by an SLX4-associated SSE (e.g. MUS81-EME1) (brown arrow) to permit D-loop formation via the invasion of the adjacent intact duplex. DNA synthesis from the invaded strand (now the nascent leading stand) might be initiated by Pol ξ and then extended by Pol δ, following a POLD3-mediated polymerase switch. This switch would facilitate the development of more extensive and processive DNA synthesis. **d** In the previously proposed 'symmetric fork cleavage' model (SFC)[1], two converging replication forks are each cleaved on the leading strand template by an SLX4-associated SSE (brown arrows). The two broken ends (encircled by green dotted lines) could be ligated by an end joining pathway, while the intact strand (encircled by orange dotted lines; using the left side as an example) could be repaired by REV1/REV3-mediated gap filling that would require Pol δ if the tract of DNA synthesis was extensive (again mediated by a POLD3-dependent polymerase switch). In (**c**) and (**d**), red dotted lines indicate new DNA synthesis catalyzed by Pol ξ and/or Pol δ in mitosis, and the blue dotted lines indicate DNA synthesis that had occurred prior to mitosis. Solid black lines denote parental DNA strands.

Reference

1. Wu, R.A., Pellman, D.S. & Walter, J.C. The Ubiquitin Ligase TRAIP: Double-Edged Sword at the Replisome. *Trends Cell Biol* **31**, 75-85 (2021).

REAGENT or RESOURCE

Antibodies	SOURCE	IDENTIFIER	Dilution factors (WB)	Dilution factors (IF)
Mouse anti-FANCD2	Santa Cruz	Cat#sc-20022, RRID: AB_2278211	/	1/200
Rabbit anti-FANCD2	Novus	Cat#NB100-182, RRID: AB_1108498	/	1/400
Mouse anti-53BP1(6B3E10)	Santa Cruz	Cat#sc-517281, RRID:AB_2921289	/	1/100
Mouse anti-Human DNA pol delta cat (A-9)	Santa Cruz	Cat# sc-17776, RRID:AB_675487	1/200	1/100
Rabbit anti-POLD1	Bethyl	Cat# A304-005A, RRID:AB_2620353	1/1000	/
Rabbit anti-POLD1 [EPR15118]	Abcam	Cat#ab186407, RRID:AB_2921290	1/1000	/
Rabbit anti-RPA	Made in-house	PMID: 32075739	/	1/1000
Rabbit anti-flag	Sigma-Aldrich	Cat# F7425, RRID:AB_439687	1/1000	/
Mouse anti-REV1 (A-11)	Santa Cruz	Cat# sc-393022, RRID:AB_2885169	1/200	1/100
Mouse anti-Actin	Sigma-Aldrich	Cat# A3853, RRID:AB_262137	1/1000	/
Mouse anti-α-Tubulin	Sigma-Aldrich	Cat# T9026, RRID:AB_477593	1/1000	/
Rabbit anti-GAPDH	Sigma-Aldrich	Cat#PLA0125, RRID:AB_2910561	1/1000	/
Mouse anti-POL H (B-7)	Santa Cruz	Cat# sc-17770, RRID:AB_2167007	1/200	/
Mouse anti-PCNA(PC10)	Santa Cruz	Cat# sc-56, RRID:AB_628110	1/200	1/100
Rabbit anti-Ubiquityl-PCNA (Lys164) (D5C7P)	Cell Signaling Technology	Cat# 13439, RRID:AB_2798219	1/1000	/
Mouse anti-Histone H3	Abcam	Cat# ab1791, RRID:AB_302613	1/1000	/
Rabbit anti-Histone H3,phospho (Ser10)	Abcam	Cat# ab5176, RRID:AB_304763	1/1000	1/1000
Rabbit anti-gammaH2AX	ThermoFisher Scientific	Catalog # MA5-33062, RRID:AB_2810155	1/1000	1/1000
Rabbit anti-RAD18	Bethyl	Cat# A301-340A, RRID:AB_937974	1/1000	/
Mouse anti-Cdt2 (B-8)	Santa Cruz	Cat# sc-166735, RRID:AB_2261795	1/200	/
Mouse anti-MAD2B / Rev7	BD Biosciences	Cat# 612266, RRID:AB_399583	1/1000	/
Mouse anti-Human POLD3	Abnova	Cat# H00010714-M01, RRID:AB_606803	1/1000	/
Guinea pig anti-PICH	Made in-house	PMID: 26633632	/	1/400
Rabbit anti-Cyclin E	Abcam	Cat# ab33911, RRID:AB_731787	1/1000	/
Anti-mouse IgG, HRP-linked Antibody	Sigma-Aldrich	Cat# A4416, RRID:AB_258167	1/3000	/
Anti-rabbit IgG, HRP-linked Antibody	Sigma-Aldrich	Cat# A6667, RRID:AB_258307	1/3000	/
Goat anti-Mouse IgG (H+L) Highly Cross-Adsorbed Secondary Antibody, Alexa Fluor 488	ThermoFisher Scientific	Cat# A-11029, RRID:AB_2534088	/	1/500
Goat anti-Rabbit IgG (H+L) Highly Cross-adsorbed Antibody, Alexa Fluor 568 Conjugated	ThermoFisher Scientific	Cat# A-11036, RRID:AB_10563566	/	1/500
Goat anti-Rabbit IgG (H+L) Highly Cross-Adsorbed Secondary Antibody, Alexa Fluor Plus 488	ThermoFisher Scientific	Cat# A32731, RRID:AB_2633280	/	1/500
Goat anti-Rabbit IgG (H+L) Cross-Adsorbed Secondary Antibody, Alexa Fluor 568	ThermoFisher Scientific	Cat# A-11011, RRID:AB_143157	/	1/500
Goat anti-Guinea Pig IgG (H+L) Highly Cross-Adsorbed Secondary Antibody, Alexa Fluor 568	ThermoFisher Scientific	Cat# A-11075, RRID:AB_2534119	/	1/500
Goat anti-Guinea Pig IgG (H+L) Highly Cross-Adsorbed Secondary Antibody, Alexa Fluor 488	ThermoFisher Scientific	Cat# A-11073, RRID:AB_2534117	/	1/500

Chemicals, Peptides, and Recombinant Proteins	SOURCE	IDENTIFIER
5-Ph-IAA	BioAcademia	Cat#30-003
Aphidicolin	Sigma-Aldrich	Cat#A0781
BamH I	New England Biolabs	Cat#R3136S
Benzonase® Nuclease	Sigma-Aldrich	Cat#E1014
BSA, Bovine Serum Albumin	Sigma-Aldrich	Cat#A7906
Cell Extraction Buffer	ThermoFisher Scientific	Cat#FNN0011
Cisplatin	Sigma-Aldrich	Cat#232120
cOmplete™ ULTRA Tablets, Mini, EASYpack Protease Inhibitor Cocktail	Sigma-Aldrich	Cat#05892970001
Doxycycline hyclate	Sigma-Aldrich	Cat#D9891
FuGENE® HD Transfection Reagent	Promega	Cat#E2311
Geneticin™ Selective Antibiotic (G418 Sulfate)	ThermoFisher Scientific	Cat#10131027
Hygromycin B	ThermoFisher Scientific	Cat#10687010
Kpn I	New England Biolabs	Cat#R3142S
Lipofectamine™ RNAiMAX Transfection Reagent	ThermoFisher Scientific	Cat#13778-150
Opti-MEM® I Reduced Serum Medium, no phenol red	ThermoFisher Scientific	Cat#11058021
PhosSTOP™	Sigma-Aldrich	Cat#4906837001
PMSF, Phenylmethylsulfonyl fluoride	Sigma-Aldrich	Cat#10837091001
Puromycin	ThermoFisher Scientific	Cat#A1113802
RIPA Lysis and Extraction Buffer	ThermoFisher Scientific	Cat#89900
RO3306	ApexBio	Cat#A8885
SDS	Sigma-Aldrich	Cat#151-21-3
T4 DNA Ligase	New England Biolabs	Cat#M0202S
Triton X-100	Sigma-Aldrich	Cat#T8787
**Critical Commercial Assays**	**SOURCE**	**IDENTIFIER**
MycoAlert™ Mycoplasma Detection Kit	Lonza	Cat#LT07-318
illustra™ RNAspin Mini RNA Isolation Kit	Sigma-Aldrich	Cat#GE25-0500-71
Click-iT® EdU Alexa Fluor® 594 Imaging Kit	ThermoFisher Scientific	Cat#C10339
QuikChange II XL Site-Directed Mutagenesis Kit	Agilent Technologies	Cat#200522
SuperScript™ III Reverse Transcriptase	ThermoFisher Scientific	Cat#18080044
**Experimental Models: Cell Lines**	**SOURCE**	**IDENTIFIER**
U2OS	ATCC	ATCC ® HTB-96
Flp-In T-REx U2OS	Mailand group, Univ. of Copenhagen	/
Flp-In T-REx U2OS, BirA*-POLD3 WT	Made in-house	/
Flp-In T-REx U2OS, BirA*-POLD3 (F238F239/A238A239)	Made in-house	/
HeLa	ATCC	ATCC ® CCL-2
HCT116	ATCC	ATCC® CCL-247
HCT116 cells expressing OsTIR1(F74G) and POLD1-mAID-Clover	Made in-house	/
U2OS-Cyclin E	Halazonetis group, Univ. Of Geneva	PMID: 29466339
U2OS-StrepHA-PCNA(WT)	Mailand group, Univ. of Copenhagen	PMID: 26711499
U2OS-StrepHA-PCNA(K164R)	Mailand group, Univ. of Copenhagen	PMID: 26711499
Isogenic pair of U2OS cell lines with REV7 or REV7-/-	D'Andrea group , Harvard Medical School, USA	PMID: 31915374
**Oligonucleotides**	**SOURCE**	**IDENTIFIER**
**SiRNAs**		
siControl: ON-TARGETplus Non-targeting Pool	Dharmacon	Cat#D-001810-10-20
siREV1-2: 5′-CAGCGCAUCUGUGCCAAAGAATT-3′	synthesized by Sigma-Aldrich	PMID: 21926160
siREV1-4: 5′-AUCGGUGGAAUCGGUUUGGAATT-3′	synthesized by Sigma-Aldrich	PMID: 21926160
siREV1-B: 5′-AAGCAUCAAAGCUGGACGACUTT-3′	synthesized by Sigma-Aldrich	PMID: 20028736

siREV7-1: 5′-GUGGAAGAGCGCGCUCAUAAATT-3′	synthesized by Sigma-Aldrich	PMID: 21926160
siREV7-3: 5′-AAGAUGCAGCUUUACGUGGAATT-3′	synthesized by Sigma-Aldrich	PMID: 21926160
siREV3-1: 5′-CGGGAUGUAGUCAAACUGCAATT-3′	synthesized by Sigma-Aldrich	PMID: 21926160
siREV3-2: 5′-CCCACUGGAAUUAAUGCACAATT-3′	synthesized by Sigma-Aldrich	PMID: 21926160
siREV3 3'UTR (Sequence starting position: 10016)	Sigma-Aldrich	/
siPOLH: 5'-CTGGTTGTGAGCATTCGTGTA -3′	synthesized by Sigma-Aldrich	PMID: 20028736
siRAD18-3: 5′-GAGCAUGGAUUAUCUAUUCAATT-3′	synthesized by Sigma-Aldrich	PMID: 21926160
siPCNA 3′UTR: 5′-AGAAUAAAGUCCAAAGUCATT-3′	synthesized by Sigma-Aldrich	PMID: 29051491
siUBC9-1: 5′-CAGGUGGAACUAAGGGACUUUTT-3′	synthesized by Sigma-Aldrich	PMID: 22366399
siUBC9-2: 5′-AAGUAGCUGUCCCAACAAAGATT-3′	synthesized by Sigma-Aldrich	PMID: 22366399
siCDT2-1: 5′-GAAUUAUACUGCUUAUCGATT-3′	synthesized by Sigma-Aldrich	PMID: 18794347
siCDT2-2: 5′-AUACAAGAGUGACUCUAUATT-3′	synthesized by Sigma-Aldrich	PMID: 18794347
**Primers**		
REV3L Forward: 5′-TGAGTTCAAATTTGGCTGTACCT-3′	synthesized by Sigma-Aldrich	PMID: 22028621
REV3L Reverse: 5′-TCTAGTCTTCAAAATTTCTTCAAGCA-3′	synthesized by Sigma-Aldrich	PMID: 22028621
GAPDH Forward: 5′-AAGGTCGGAGTCAACGGATTTGGT-3′	synthesized by Sigma-Aldrich	PMID: 20028736
GAPDH Reverse: 5′-AGTGATGGCATGGACTGTGGTCAT-3′	synthesized by Sigma-Aldrich	PMID: 20028736
POLD3.KpnIF: 5′-TAAGGTACCATGGCGGACCAG-3′	synthesized by Sigma-Aldrich	designed -in-house
POLD3.BamHI: 5′-CTAGGATCCTTATTTCCTCTGGAAG-3′	synthesized by Sigma-Aldrich	designed -in-house
POLD3 (F238A/F239A) F: 5′-GAATATGATGAGCAACGCTGCTGGAAAAGCTGCTATG-3′	synthesized by Sigma-Aldrich	designed -in-house
POLD3 (F238A/F239A) R: 5′-CATAGCAGCTTTTCCAGCAGCGTTGCTCATCATATTC-3′	synthesized by Sigma-Aldrich	designed -in-house
REV3L-ATA-F : 5′-GCTAGGGTTGTATATGGCGcTACTGCCAGTATGTTTGTGCTACTG-3′	synthesized by Sigma-Aldrich	PMID: 27481099
REV3L-ATA-R: 5′-CAGTAGCACAAACATACTGGCAGTAgCGCCATATACAACCCTAGC-3′	synthesized by Sigma-Aldrich	PMID: 27481099
**Recombinant DNA**		
pcDNA5_FRT-TO_HA-BirA*	Mailand group, Univ. of Copenhagen	/
pOG44	ThermoFisher Scientific	Cat#V600520
pCDNA5_FRT-TO_HA-BirA*-POLD3 WT	Made in-house	/
pCDNA5_FRT-TO_HA-BirA*-POLD3 (F238F239/A238A239)	Made in-house	/
pEF6/V5-REV3-3xFLAG	Canman group, Univ. of Michigan	PMID: 21926160
pEF6/V5-REV3-3xFLAG-ATD/ATA	Made in-house	/
**Software and Algorithms**	**SOURCE**	
ImageJ	NIH	
Prism 9.0.0	GraphPad	
Olympus cellSens Software	Olympus	
TIBCO Spotfire	PerkinElmer	
FlowJO	BD Biosciences	

Gating strategy used in flow cytometry analysis (Methods)

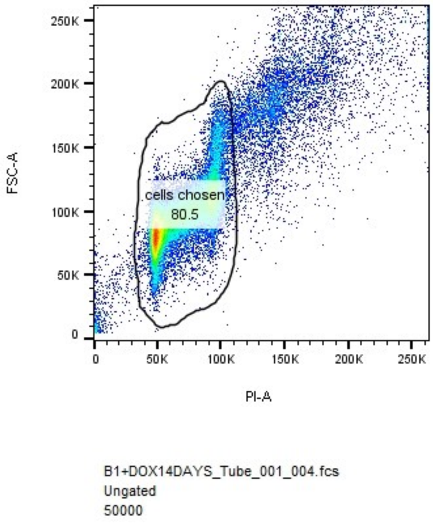

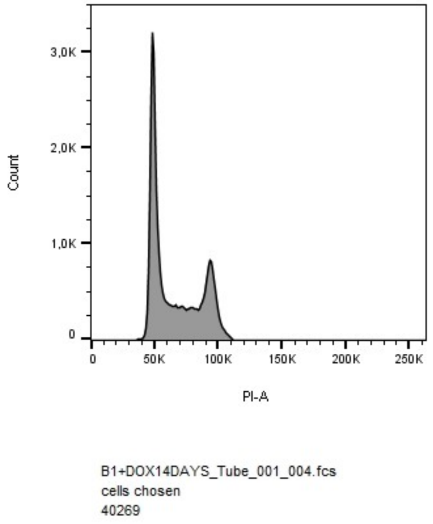

Above left, a representative a chart of of untreated U2OS gated on a forward scatter (FSC-A) / total PI fluorescence (PI-A) plot. In this chart, the black circle indicated the boundary of gating single cells. Right, singlets (80-90%) were represented as cell count / PI fluorescence(PI-A) in a histogram. 50,000 cells were analysed in each condition.
