# Supplementary material for: Mitotic DNA synthesis in response to replication stress requires the sequential action of DNA polymerases zeta and delta in human cells
Source: Nat Commun. 2023 Feb 9;14:706. doi: 10.1038/s41467-023-35992-5 (PMC9911744; doi:10.1038/s41467-023-35992-5)
Supplement: Supplementary file 3 — Reporting Summary [file 41467_2023_35992_MOESM3_ESM.pdf]

nature portfolio

Corresponding author(s): Wei Wu, Ying Liu

Last updated by author(s): Dec 27, 2022

Reporting Summary

Nature Portfolio wishes to improve the reproducibility of the work that we publish. This form provides structure for consistency and transparency in reporting. For further information on Nature Portfolio policies, see our Editorial Policies and the Editorial Policy Checklist.

Statistics

For all statistical analyses, confirm that the following items are present in the figure legend, table legend, main text, or Methods section.

n/a	Confirmed	
☐	☒	The exact sample size (*n*) for each experimental group/condition, given as a discrete number and unit of measurement
☐	☒	A statement on whether measurements were taken from distinct samples or whether the same sample was measured repeatedly
☐	☒	The statistical test(s) used AND whether they are one- or two-sided<br>*Only common tests should be described solely by name; describe more complex techniques in the Methods section.*
☒	☐	A description of all covariates tested
☒	☐	A description of any assumptions or corrections, such as tests of normality and adjustment for multiple comparisons
☐	☒	A full description of the statistical parameters including central tendency (e.g. means) or other basic estimates (e.g. regression coefficient) AND variation (e.g. standard deviation) or associated estimates of uncertainty (e.g. confidence intervals)
☐	☒	For null hypothesis testing, the test statistic (e.g. *F*, *t*, *r*) with confidence intervals, effect sizes, degrees of freedom and *P* value noted<br>*Give P values as exact values whenever suitable.*
☒	☐	For Bayesian analysis, information on the choice of priors and Markov chain Monte Carlo settings
☒	☐	For hierarchical and complex designs, identification of the appropriate level for tests and full reporting of outcomes
☒	☐	Estimates of effect sizes (e.g. Cohen's *d*, Pearson's *r*), indicating how they were calculated

Our web collection on statistics for biologists contains articles on many of the points above.

Software and code

Policy information about availability of computer code

Data collection	n/a
Data analysis	ImageJ NIH<br>Prism 9.0.0 GraphPad<br>Olympus cellSens Software Olympus<br>TIBCO Spotfire PerkinElmer<br>FlowJO BD Biosciences

For manuscripts utilizing custom algorithms or software that are central to the research but not yet described in published literature, software must be made available to editors and reviewers. We strongly encourage code deposition in a community repository (e.g. GitHub). See the Nature Portfolio guidelines for submitting code & software for further information.

Data

Policy information about availability of data

All manuscripts must include a data availability statement. This statement should provide the following information, where applicable:

- Accession codes, unique identifiers, or web links for publicly available datasets
- A description of any restrictions on data availability
- For clinical datasets or third party data, please ensure that the statement adheres to our policy

Provide your data availability statement here.

Human research participants

Policy information about studies involving human research participants and Sex and Gender in Research.

Reporting on sex and gender	n/a
Population characteristics	n/a
Recruitment	n/a
Ethics oversight	n/a

Note that full information on the approval of the study protocol must also be provided in the manuscript.

Field-specific reporting

Please select the one below that is the best fit for your research. If you are not sure, read the appropriate sections before making your selection.

☒ Life sciences ☐ Behavioural & social sciences ☐ Ecological, evolutionary & environmental sciences

For a reference copy of the document with all sections, see nature.com/documents/nr-reporting-summary-flat.pdf

Life sciences study design

All studies must disclose on these points even when the disclosure is negative.

Sample size	No statistical method was used to predict sample size. The sample size was guided by preliminary studies we carried out, and guided by a number of previous publications using similar approaches. Sample size was kept similar amongst different conditions. In assays related to fluorescence of mitotic cells, we collected 30-55 for each condition to generate robust statistical data. All cells imaged during any experiment were quantified. For colony formation assays, all of the colonies stained were counted
Data exclusions	No data were excluded from each analysis.
Replication	Each data set in cellular biology experiments were generated from three independent experiments.
Randomization	n/a
Blinding	In all of the cellular assays, in the steps of counting immunofluorescence foci, ultra fine DNA bridges (UFBs), or colonies fixed in plates, the samples were coded and analyzed blindly.

Reporting for specific materials, systems and methods

We require information from authors about some types of materials, experimental systems and methods used in many studies. Here, indicate whether each material, system or method listed is relevant to your study. If you are not sure if a list item applies to your research, read the appropriate section before selecting a response.

Materials & experimental systems

n/a	Involved in the study
	☒ Antibodies
	☒ Eukaryotic cell lines
☒	Palaeontology and archaeology
☒	Animals and other organisms
☒	Clinical data
☒	Dual use research of concern

Methods

n/a	Involved in the study
☒	ChIP-seq
	☒ Flow cytometry
☒	MRI-based neuroimaging

Antibodies

Antibodies used

Mouse anti-FANCD2 Santa Cruz Cat#sc-20022, RRID: AB_2278211, IF, 1/200
Rabbit anti-FANCD2 Novus Cat#NB100-182, RRID: AB_1108498, IF, 1/400
Mouse anti-53BP1(6B3E10) Santa Cruz Cat#sc-517281, RRID:AB_2921289, IF,1/100
Mouse anti-Human DNA pol delta cat (A-9) Santa Cruz Cat# sc-17776, RRID:AB_675487, WB, 1/200; IF,1/100
Rabbit anti-POLD1 Bethyl Cat# A304-005A, RRID:AB_2620353 , WB, 1/1000
Rabbit anti-POLD1 [EPR15118] Abcam Cat#ab186407, RRID:AB_2921290 , WB, 1/1000
Rabbit anti-RPA Made in-house PMID: 32075739, IF, 1/1000
Rabbit anti-flag Sigma-Aldrich Cat# F7425, RRID:AB_439687 , WB, 1/1000
Mouse anti-REV1 (A-11) Santa Cruz Cat# sc-393022, RRID:AB_2885169, WB, 1/200; IF, 1/100
Mouse anti-Actin Sigma-Aldrich Cat# A3853, RRID:AB_262137 , WB, 1/1000
Mouse anti-α-Tubulin Sigma-Aldrich Cat# T9026, RRID:AB_477593 , WB, 1/1000
Rabbit anti-GAPDH Sigma-Aldrich Cat#PLA0125, RRID:AB_2910561 , WB, 1/1000
Mouse anti-POL H (B-7) Santa Cruz Cat# sc-17770, RRID:AB_2167007 , WB, 1/200
Mouse anti-PCNA(PC10) Santa Cruz Cat# sc-56, RRID:AB_628110 , WB, 1/200; IF1/100
Rabbit anti-Ubiquityl-PCNA (Lys164) (D5C7P) Cell Signaling Technology Cat# 13439, RRID:AB_2798219 , WB, 1/1000
Mouse anti-Histone H3 Abcam Cat# ab1791, RRID:AB_302613 , WB, 1/1000
Rabbit anti-Histone H3,phospho (Ser10) Abcam Cat# ab5176, RRID:AB_304763 , WB, 1/1000; IF1/1000
Rabbit anti-gammaH2AX ThermoFisher Scientific Catalog # MA5-33062, RRID:AB_2810155, WB, 1/1000; IF, 1/1000
Rabbit anti-RAD18 Bethyl Cat# A301-340A, RRID:AB_937974 , WB, 1/1000
Mouse anti-Cdt2 (B-8) Santa Cruz Cat# sc-166735, RRID:AB_2261795 , WB, 1/200
Mouse anti-MAD2B / Rev7 BD Biosciences Cat# 612266, RRID:AB_399583, WB, 1/1000
Mouse anti-Human POLD3 Abnova Cat# H00010714-M01, RRID:AB_606803, WB, 1/1000
Guinea pig anti-PICH Made in-house PMID: 26633632, IF, 1/400
Rabbit anti-Cyclin E Abcam Cat# ab33911, RRID:AB_731787, WB, 1/1000
Anti-mouse IgG, HRP-linked Antibody Sigma-Aldrich Cat# A4416, RRID:AB_258167 , WB, 1/3000
Anti-rabbit IgG, HRP-linked Antibody Sigma-Aldrich Cat# A6667, RRID:AB_258307 , WB, 1/3000
Goat anti-Mouse IgG (H+L) Highly Cross-Adsorbed Secondary Antibody, Alexa Fluor 488 ThermoFisher Scientific Cat# A-11029, RRID:AB_2534088, IF, 1/500
Goat anti-Rabbit IgG (H+L) Highly Cross-adsorbed Antibody, Alexa Fluor 568 Conjugated ThermoFisher Scientific Cat# A-11036, RRID:AB_10563566 , IF, 1/500
Goat anti-Rabbit IgG (H+L) Highly Cross-Adsorbed Secondary Antibody, Alexa Fluor Plus 488 ThermoFisher Scientific Cat# A32731, RRID:AB_2633280, IF, 1/500
Goat anti-Rabbit IgG (H+L) Cross-Adsorbed Secondary Antibody, Alexa Fluor 568 ThermoFisher Scientific Cat# A-11011, RRID:AB_143157, IF, 1/500
Goat anti-Guinea Pig IgG (H+L) Highly Cross-Adsorbed Secondary Antibody, Alexa Fluor 568 ThermoFisher Scientific Cat# A-11075, RRID:AB_2534119, IF, 1/500
Goat anti-Guinea Pig IgG (H+L) Highly Cross-Adsorbed Secondary Antibody, Alexa Fluor 488 ThermoFisher Scientific Cat# A-11073, RRID:AB_2534117, IF, 1/500

Validation

n/a

Eukaryotic cell lines

Policy information about cell lines and Sex and Gender in Research

Cell line source(s)

U2OS, ATCC ® HTB-96
Flp-In T-REx U2OS, Mailand group, Univ. of Copenhagen
Flp-In T-REx U2OS, BirA*-POLD3 WT, Made in-house
Flp-In T-REx U2OS, BirA*-POLD3 (F238F239/A238A239) Made in-house
HeLa, ATCC ® CCL-2
HCT116, ATCC® CCL-247
HCT116 cells expressing OsTIR1(F74G) and POLD1-mAID-Clover, Made in-house
U2OS-Cyclin E Halazonetis group, Univ. Of Geneva, PMID: 29466339
U2OS-StrepHA-PCNA(WT) Mailand group, Univ. of Copenhagen, PMID: 26711499
U2OS-StrepHA-PCNA(K164R) Mailand group, Univ. of Copenhagen, PMID: 26711499
Isogenic pair of U2OS cell lines with REV7 or REV7-/- D'Andrea group , Harvard Medical School, USA, PMID: 31915374

Authentication

U2OS, ATCC ® HTB-96, HeLa, ATCC ® CCL-2, HCT116, ATCC® CCL-247 cells were authenticated by STR profiling. For the cell

Authentication	lines generated by us or collaborators, including Flp-In T-REx U2OS, Flp-In T-REx U2OS, Flp-In T-REx U2OS BirA*-POLD3 WT/ BirA*-POLD3 (F238F239/A238A239), HCT116 cells expressing OsTIR1(F74G) and POLD1-mAID-Clover, U2OS-StrepHA-PCNA(WT) or (K164R), U2OS cell lines with REV7 or REV7-/-, we authenticate the cell lines by their morphology, doubling time, and where possible, testing at least two clones in our assays.
Mycoplasma contamination	All of cells were subjected to monthly mycoplasma testing (using MycoAlert kit; Lonza). Only mycoplasma-free cells were analyzed.
Commonly misidentified lines<br>(See ICLAC register)	n/a

Flow Cytometry

Plots

Confirm that:

☒ The axis labels state the marker and fluorochrome used (e.g. CD4-FITC).

☒ The axis scales are clearly visible. Include numbers along axes only for bottom left plot of group (a 'group' is an analysis of identical markers).

☒ All plots are contour plots with outliers or pseudocolor plots.

☒ A numerical value for number of cells or percentage (with statistics) is provided.

Methodology

Sample preparation	Cells were harvested by trypsinization and fixed by drop wise addition of ice-cold 70% ethanol on the cell pellet while gently vortexing, followed by incubation at -20 °C for overnight. Fixed cells were centrifuged at 2,000g for 20 mins at 4°C and the cell pellet was washed two times with 1× PBS (500 g X 5 min). Following that, the cells were stained for 30 mins at 37°C with 80 µg/ml propidium iodide and 10 mg/ml RNase A dissolved in 1X PBS.
Instrument	FACSCelesta
Software	FACSDiva
Cell population abundance	There was no sorting involved.
Gating strategy	For all experiments, cells were gated according to forward scatter (FSC-A) / PI-Area (PI fluorescence) to analyze only singlets.

☒ Tick this box to confirm that a figure exemplifying the gating strategy is provided in the Supplementary Information.
