# Supplementary material for: Mitotic DNA synthesis in response to replication stress requires the sequential action of DNA polymerases zeta and delta in human cells
Source: Nat Commun. 2023 Feb 9;14:706. doi: 10.1038/s41467-023-35992-5 (PMC9911744; doi:10.1038/s41467-023-35992-5)

Source Data of Western blots -1

Figure 1c

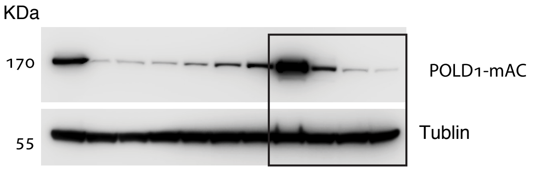

Figure 1e

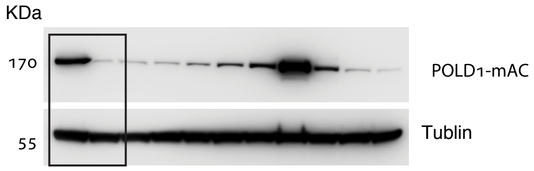

Figure 1i

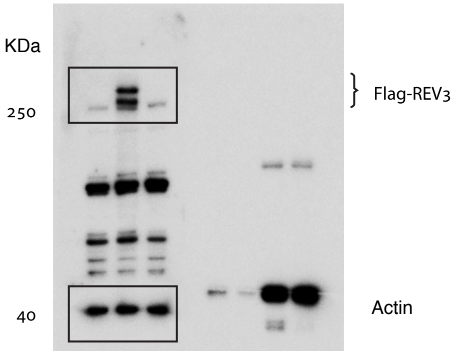

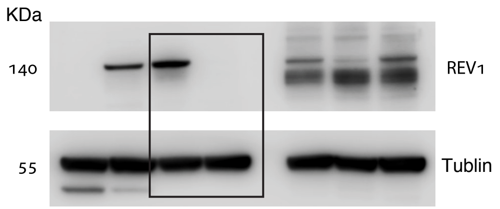

Figure 2b

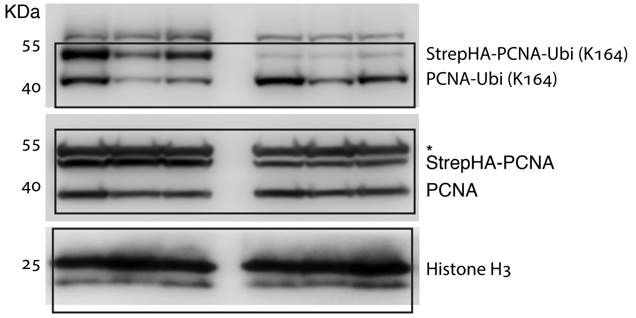

Figure 2d

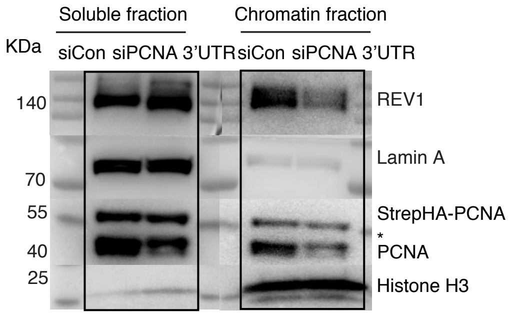

StrepHA-PCNA K164R U2OS prometaphase cells

Figure 2h

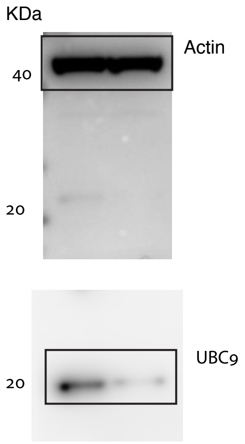

Figure 3b

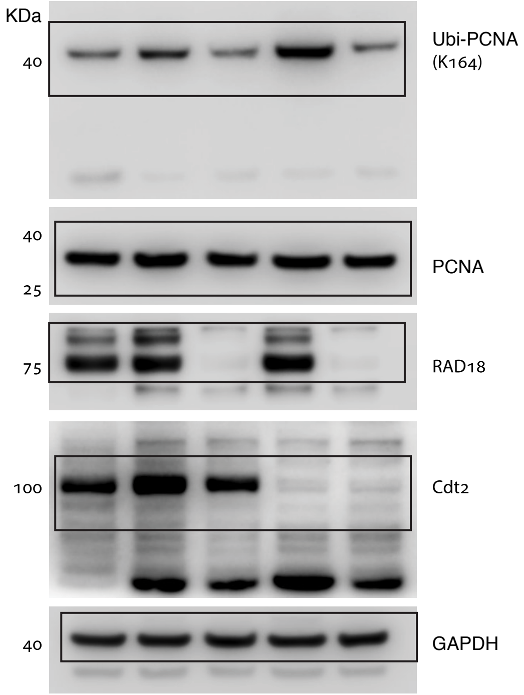

Source Data of Western blots -2

Figure 4g

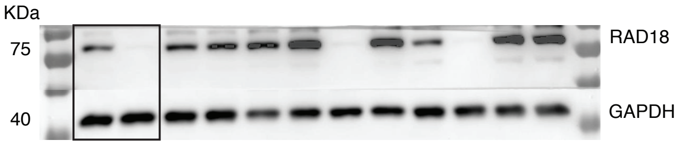

Figure 5c

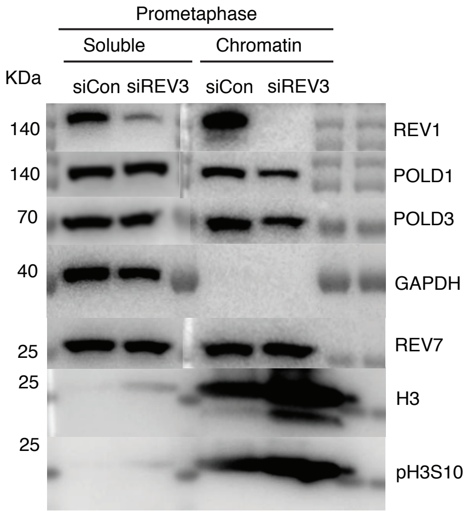

Figure 5d

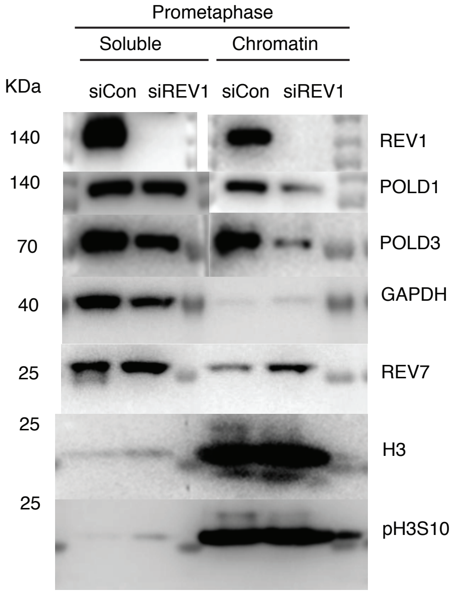

Figure 5e

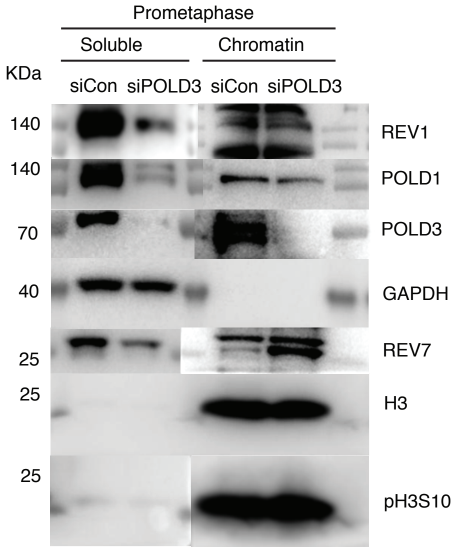

Figure 5f

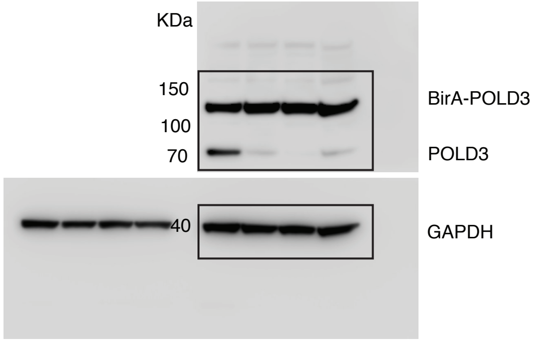

Figure 5h

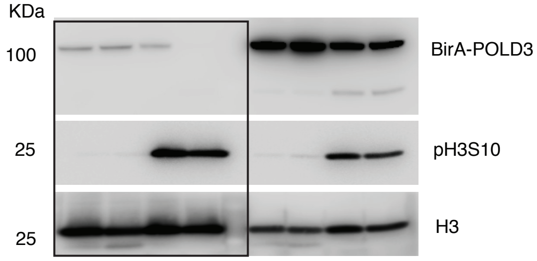

Source Data of Western blots-3

Figure 6c

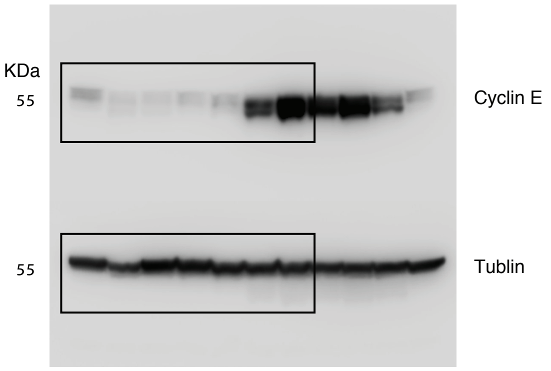

Figure 6g

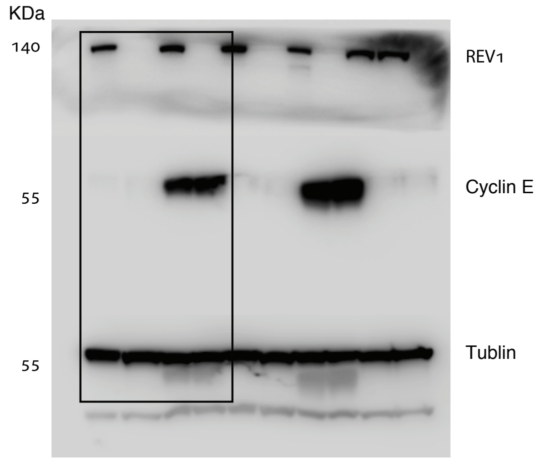

Figure 7c

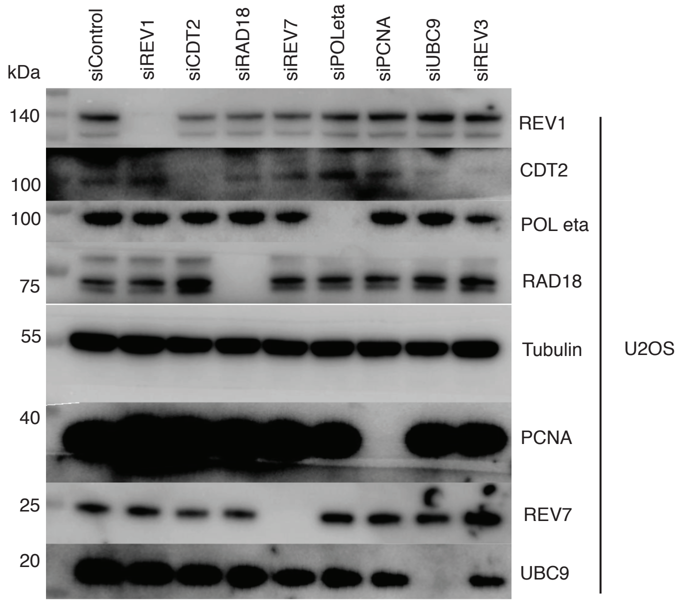

Figure 7f

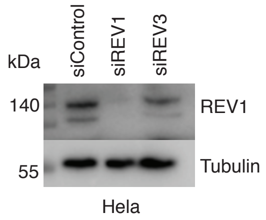

Source Data of Western blots-4

Figure S1b

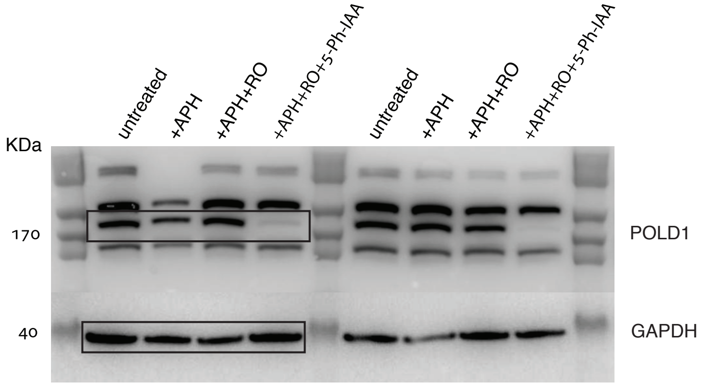

Figure S2c

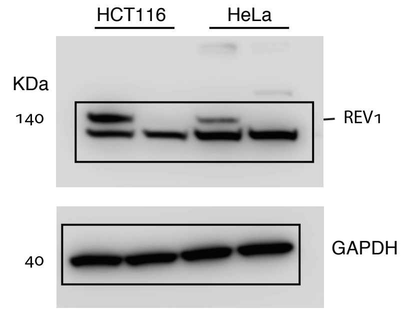

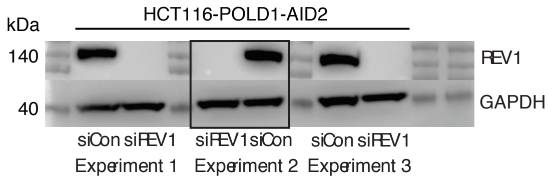

Figure S5b

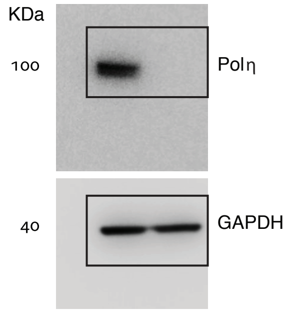

Figure S 7e

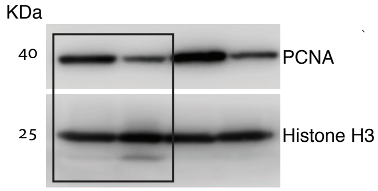

Figure S9b

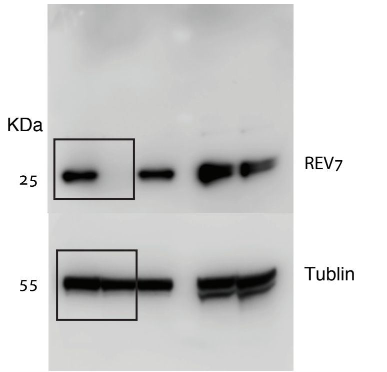

Figure S8b

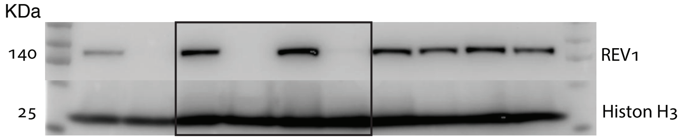

Figure S9f

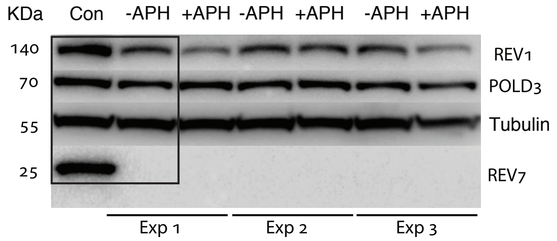

Figure S10b

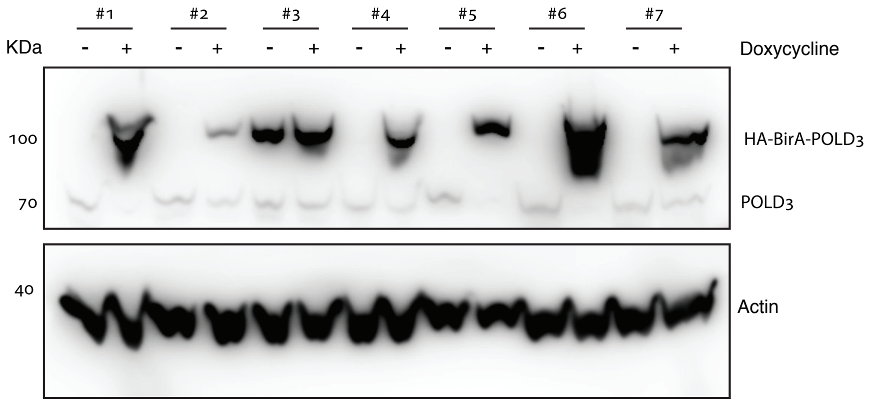

Figure S11b

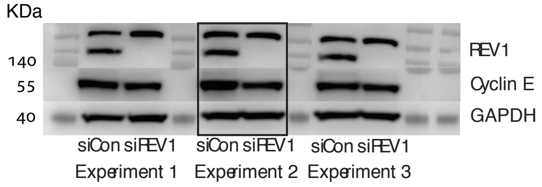

Supplement: Supplementary file 4 — Source Data [file 41467_2023_35992_MOESM4_ESM.zip › Source data for western blots.pdf]
